# Test–retest reliability of lower limb muscle strength, jump and sprint performance tests in elite female team handball players

**DOI:** 10.1007/s00421-024-05470-x

**Published:** 2024-04-09

**Authors:** Bjørn Fristrup, Peter Krustrup, Kevin Højer Kristensen, Steffen Rasmussen, Per Aagaard

**Affiliations:** 1 Department of Sports Science and Clinical Biomechanics, Research Unit for Muscle Physiology and Biomechanics (MoB), University of Southern Denmark, Odense, Denmark; 2 Department of Sports Science and Clinical Biomechanics, SDU Sport and Health Sciences Cluster (SHSC), University of Southern Denmark, Odense, Denmark; 3 Danish Institute for Advanced Study (DIAS), University of Southern Denmark, Odense, Denmark; 4 Sport and Health Sciences, University of Exeter, Exeter, UK

**Keywords:** Reproducibility, Mechanical muscle function, Explosive muscle strength, Vertical jump, Power, Acceleration

## Abstract

**Purpose:**

This study aimed to assess the reliability of lower limb muscle function (knee extensor/flexor peak torque, rate of torque development (RTD), impulse, and countermovement jump (CMJ) performance) and sprint performance (acceleration capacity).

**Methods:**

CMJ performance was evaluated on a force plate. MVIC, RTD and impulse variables were investigated using a portable isometric dynamometer and sprint performance was assessed with dual-beam photocells in elite female athletes.

**Results:**

CMJ test variables maximal vertical jump height, peak and mean power, concentric work, and body center of mass displacement demonstrated good-to-excellent test–retest correlations between Test 1 and Test 2 (ICC ≥ 0.70, CW_w-s_ = 3.4–11.0%). Peak MVIC torque for the knee extensors and flexors demonstrated excellent test–retest correlations (both ICC = 0.84) along with CV_w-s_ values of 6.8 and 6.0%, respectively. Late-phase (0–100 ms, 0–200 ms) RTD for the knee flexors demonstrated excellent test–retest correlations (ICC = 0.89–0.91, CV_w-s_ = 4.8–8.5%). Sprint times at 10- and 20-m demonstrated excellent test–retest reproducibility (ICC = 0.83 and ICC = 0.90, respectively) with CV_w-s_ values of 1.9 and 1.5%. For 5-m sprint times, test–retest reproducibility was good (ICC = 0.71) with CV_w-s_ of 2.8%. Sprint testing performed while dribbling a handball improved (*p* < 0.05) from test to retest at 5-, 10- and 20-m.

**Conclusion:**

In conclusion, the force plate, the mobile isometric dynamometer, and dual-beam photocells provide reproducible tools for field-based testing of countermovement jump performance, knee extensor and flexor strength and sprint performance.

## Introduction

Physiological testing in elite athletes requires reliable measurement tools, which can provide accurate and reproducible information about physical performance capacity (Svensson and Drust 2005; Bangsbo et al. 2006; Chirosa Ríos et al. 2022). Even minor improvements in mechanical muscle function (down to 1%) can make a significant difference for team sport players (Currell and Jeukendrup 2008), which means that precise test equipment and reproducible measurement protocols are required to assess the magnitude of training-induced adaptations in various elite athlete populations.

Elite athletes typically face challenges in balancing their time between sports-specific training, general and specialized strength and endurance training, mental conditioning, mixed with the demands of weekly or monthly competitions (Sands 2008). Consequently, it becomes impractical to incorporate time-consuming lab-based testing. Hence, there is a need to develop reliable sports-specific field-based test tools and protocols for athletes that can be conducted at (or close to) their regular training sites (Bangsbo et al. 2006; Sands 2008; Morenas-Aguilar et al. 2023). Performing physiological testing in athletes in their local environment, therefore, may mitigate the logistical challenges associated with bringing athletes to remote University Labs or supporting facilities for physiological testing.

Female elite team handball is characterized by its physically demanding and intermittent nature, involving a significant amount of aerobic energy expenditure interspaced by bouts of anaerobic intense actions such as jumps, sprints, turns, side-cuts accelerations and decelerations (Póvoas et al. 2012, 2014; Michalsik et al. 2014; Luigi Bragazzi et al. 2020). These and other intense player actions necessitate rapid contraction abilities of the lower limbs, underscoring the importance of accurately monitoring single and multi-joint muscle function (strength, power, RFD, sprint capacity) in the lower limbs of elite team handball athletes.

Mechanical muscle function in the lower limbs can be evaluated by means of isometric dynamometry, which is typically used to measure isolated single-joint muscle strength (peak torque), and rate of torque development (RTD), where RTD (Δtorque/Δtime) can be derived from the torque–time curve obtained during maximal voluntary isometric contraction (MVIC) (Aagaard et al. 2002; Maffiuletti et al. 2007). The reliability of such maximal knee extensor and flexor strength measurements has been examined previously for various dynamometers (Biodex, Cybex, KinCom, Merac, Lido, Orthotron, Technogym, and Con-Trex) demonstrating moderate-to-high degrees of inter-session test–retest reliability (ICC = 0.89–0.98, CV 4.7–5.5%) (Tredinnick and Duncan 1988; Capranica et al. 1998; Lund et al. 2005; Maffiuletti et al. 2007). While these isokinetic/isometric devices all are expensive, heavy, and non-portable (Lab based) space-demanding equipment, portable dynamometers may be more attractive employment in situations of on-site field testing. As one such device, the S2P isometric dynamometer is a light-weight (70 kg) portable device agile for field testing. However, to our best knowledge, only limited data exist about the reproducibility (i.e., test–retest reliability) of this device, with a single study reporting strong reliability for knee extensor MVIC peak torque (ICC = 0.97–0.98, CV = 7.1–8.2%) (Sarabon et al. 2013), whereas no measures of knee flexor MVIC peak torque or explosive muscle strength (RTD, impulse) have been reported.

Team handball is a contact sport with a high risk of both contact and non-contact injuries (Vila et al. 2022). Low peak torque H/Q-ratios have previously been suggested to increase the risk of non-contact anterior cruciate ligament (ACL) injuries (Aagaard et al. 1998; Greco et al. 2012; Hannah et al. 2015). Consequently, it becomes imperative to ensure reliable measurements of H/Q strength (peak torque) ratios.

Countermovement jump (CMJ) testing represents a valuable tool for the evaluation of lower body multi-joint power production generated during rapid stretch–shortening cycle (SSC) muscle actions (Caserotti et al. 2001; Markovic et al. 2004; Bojsen-Møller et al. 2005; Thorlund et al. 2008; Jakobsen et al. 2012). A study by Heishman (2020) investigated the test–retest reliability in CMJ performance on a force plate (jump height, peak power, mean power and peak vertical ground reaction force (GRF)) in team sport athletes (22 female NCAA Division 1 collegiate basketball players, 14 men and 8 women aged 18–22 years) and found excellent intraclass correlation coefficients (ICC) accompanied by low coefficients of variation (CV) (ICC ≥ 0.80, CV ≤ 10%) (Heishman et al. 2020). Concentric peak power during CMJ testing demonstrated good-to-excellent test–retest reliability (ICC = 0.74–0.77, CV_w-s_ 8.8–10.8%) in a study by Lindberg et al. (2021), examining a mixed cohort of 27 male and female team sport athletes (team handball and ice hockey players aged 21 ± 5 years) (Lindberg et al. 2021). However, to our best knowledge, no previous study has examined the reliability of CMJ power testing in elite female team handball players.

In intermittent elite team sports such as team handball, the game format dispose for a high number of short-distance (≤ 10 m) sprints (i.e., accelerations) as well sprints performed at near maximal-to-maximal running speed (Manchado et al. 2013). Thus, precise evaluation of acceleration/sprint capacity is essential in the monitoring of functional performance in elite team sports athletes. For this purpose, dual-beam photocells have been recommended as the most accurate tool for measuring short-distance sprint times (including 5- and 10-m split times), which typically have been employed during 20-m linear sprinting from a standing start (Gabbett 2010; Haugen et al. 2014; Haugen and Buchheit 2016).

The objective of the present study was to investigate between session test–retest reliability of selected field-based testing methods applicable in the local environment of elite team sports athletes. Experimental focus was on lower limb muscle function (knee extensor/flexor peak torque, RTD, impulse, SSC (CMJ) power, lower leg stiffness) and whole-body movement performance (acceleration capacity and CMJ jump height). Specifically, the study aimed to examine the degree of test–retest reliability of various MVIC strength and RFD (RTD) measures obtained for the knee extensors and flexors using a portable isometric dynamometer. In addition, it was the aim to assess the test–retest reliability in maximal vertical jump height (CMJ) and SSC leg extensor power obtained during CMJ testing along with a similar evaluation of short-distance sprint capacity (0–5 m, 0–10 m, 0–20 m).

## Materials and methods

### Study design

**Fig. 1:**
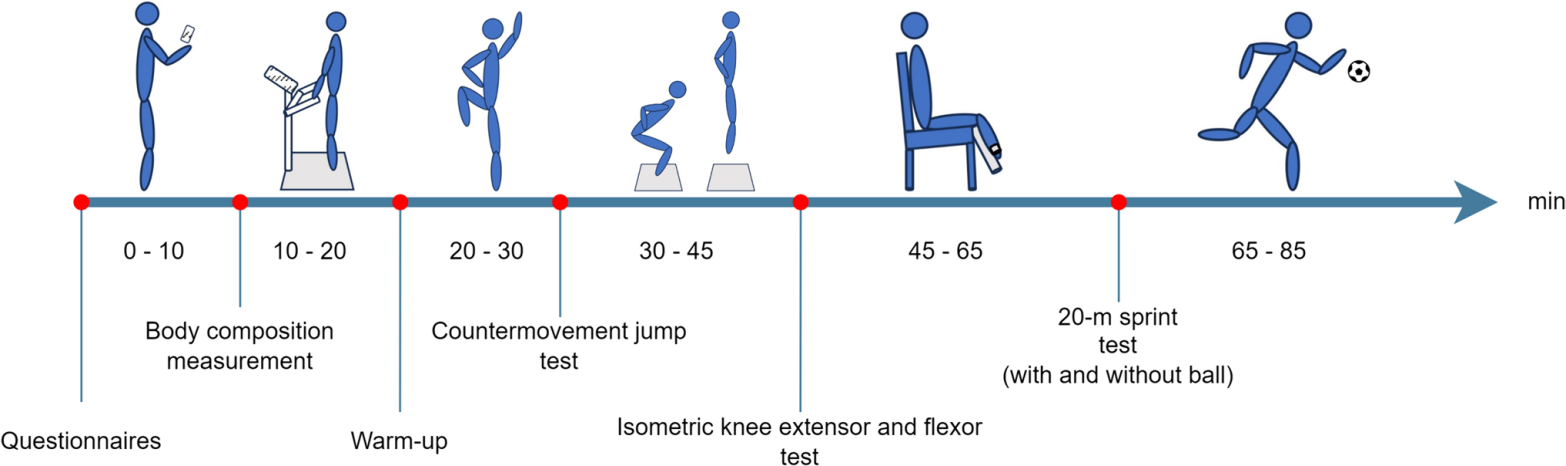
Estimated timeline for the test protocol. Study participants started by filling in an online questionnaires on their mobile device. Bodycomposition was measured followed by standardized warm-up, countermovement jump test, maximal voluntary contraction strength test of the knee extensors and flexors and lastly 20-m sprint with and without ball

Test–retest sessions (Test 1, Test 2) were separated by one week between the tests (Hopkins et al. 2001). All study participants were assessed at the same time of day, using the same order of participants and subtests. In a separate familiarization test session conducted within one month prior to Test 1, all study participants were familiarized with the entire test protocol; questionnaires, body composition measurements, warm-up, countermovement jump testing, assessments of maximal muscle strength and RTD for the knee extensors and flexors, and measurements of 20-m sprint capacity with and without dribbles with handball (see Fig. 1). All instructions and steps of data collection were managed by the same assessor at both tests. Standardized oral instructions were provided to all players before initiation of each test.

### Subjects

Sixty female youth elite team handball players from the Danish U17 and U19 league agreed to participate in the study. Twenty players completed all three test sessions (Table 1). Study participants received written and oral information about the experimental procedures prior to giving their written informed consent. Parental or guardian signed consent was obtained from subjects < 18 years. The study was registered at the Regional Committees on Health Research Ethics for Southern Denmark (20,212,000–114). Subject characteristics are listed in Table 1.

**Table 1:** Participant characteristics

	Test 1 *n* = 20 mean ± SD	Test 2 *n* = 20 mean ± SD
Age (years)	17.8 ± 1.1	17.8 ± 1.1
Height (cm)	173.4 ± 5.9	173.3 ± 5.9
Body mass (kg)	70.5 ± 8.1	70.8 ± 8.1
Skeletal muscle mass (kg)	30.7 ± 3.3	30.7 ± 3.4
Body fat percentage (%)	22.3 ± 5.0	22.3 ± 5.6
Years of playing team handball (years)		12.0 ± 1.1
Years of playing on elite level (years)		2.1 ± 0.1
Playing position (*n*):		
Goalkeepers		4
Center backs (playmakers)		2
Backs (left/right)		6
Wings (left/right)		5
Center forwards (pivots)		3

### Body composition

Bioimpedance (InBody 270, InBody Co., LTD, Seoul, South Korea) measurements were performed to assess body mass (BM), skeletal muscle mass (SMM) and fat percentage (FAT%). Body height was measured twice (Leicester Height Measure Mk II, Child Growth Foundation, Newcastle, England) and an average was calculated. Athletes were not subjected to any restrictions on energy-intake before the measurements.

### Warm-up procedure

All study participants completed a standardized ~ 10-min warm-up program. The warm-up program consisted of running, strength exercises and ball throws. Half-court dash runs; 4 × low-to-high intensity runs, 2 × sidesteps, 1 × backwards run and 2 × skip running. Body weight strength exercises were: 10 × squat, 10 × single-leg Romanian deadlift (5/5), 10 × sit-ups, 10 back extensions, 10 × push-ups, 20 × single-leg medio-lateral leg swings (10/10), 20 × single-leg anterior–posterior leg swings (10/10), 20 × forward and backward arm swings (10/10) for each arm, followed by 8 × single-leg jumps (4/4), and 8 × random change of direction movements. Lastly, participants performed 20 × ball throws (10 × medium velocity, 10 × high velocity).

### CMJ performance (SSC muscle power)

Stretch–shortening cycle (SSC) muscle performance was assessed in a bilateral countermovement jump test performed on an instrumented force plate (AccuPower, AMTI, Watertown, USA) (Caserotti et al. 2001; Bojsen-Møller et al. 2005). Two initial submaximal warm-up jumps were performed followed by five maximal single effort jumps, each interspaced by 30-s recovery. Subjects were instructed to execute the jump in a continuous movement, while focusing on jumping as high and forcefully as possible with their hands positioned on the hips (Caserotti et al. 2001; Thorlund et al. 2008; Jakobsen et al. 2012). Vertical ground reaction force (F_z_) signals were A/D converted at 1000 Hz using custom build software script (MATLAB, MathWorks, Natick, USA). During later off-line analysis, the F_z_ signals were analyzed also using custom build software script (MATLAB, MathWorks, Natick, USA), where the jump with the highest jump height was chosen for further statistical analysis. All CMJ take-offs were divided into an eccentric (downward) phase (E_p_) and a concentric (upward) phase (C_p_), the former defined as the time interval with downward body center of mass (BCM) movement to its deepest position (V = 0) and the latter defined as the upward BCM movement from its deepest position to toe-off (Caserotti et al. 2001; Thorlund et al. 2008; Jakobsen et al. 2012). E_p_ was further divided into an eccentric acceleration phase (E_p-acc_), the interval from onset of downwards movement to the instant of maximal negative (downwards) BCM velocity (V_peak_ [E_p_]), and an eccentric deceleration phase E_p-dec_, defined as the interval from V_peak_ (E_p_) to BMC deepest position (V = 0) (Caserotti et al. 2001; Thorlund et al. 2008; Jakobsen et al. 2012). Vertical jump height was calculated as ${{\text{V}}}_{{\text{to}}}^{2}/2{\text{g}}$ where V_to_ denotes the take-off velocity of BCM calculated from time integration of the net upward ground reaction force (F_z_) during the concentric take-off phase $({\text{V}}_{{{\text{to}}}} \, = \,\int {\left[ {\left( {{\text{F}}_{{\text{z}}} /{\text{BM}}} \right) - {\text{g}}} \right] \cdot {\text{d}}t)}$, BM = body mass (kg), gravitational acceleration (g = 9.81 m/s^2^) (Bojsen-Møller et al. 2005; Holsgaard Larsen et al. 2007; Jakobsen et al. 2012). Besides maximal vertical jump height, jumps were analyzed for jump height relative to ground level (JH_GL_), calculated as JH + (BCM_disp_ [C_p_] – BCM_disp_ [E_p_]), peak and mean take-off power, work exerted on BCM, rate of force development (RFD) calculated as the average tangential slope at 0–100 ms relative to the start of the E_p-dec_, duration of the concentric take-off phase (C_p_; BCM moving from deepest position to toe-off), BCM displacement (BCM_disp_) in the E_p_ and C_p_, and lower limb stiffness (LLS) calculated as LLS = ΔvGRF/ΔBCM_disp_ (E_p_), where ΔvGRF is change (Δ) in vertical (v) ground reaction force (GRF) (Jordan et al. 2023).

### Isometric knee extensor and flexor strength

MVIC peak torque was measured for the knee extensors and flexors of the dominant leg (take-off leg in jump-shooting), along with the assessment of RTD and impulse using a portable isometric dynamometer (Dynamometer, Science to Practice (S2P), Ljubljana Slovenia). Anatomical knee joint angle was (mean ± SD) 50.2 ± 3.2° and 46.3 ± 4.1° for the knee extensor and flexor MVIC tests (0° = full knee extension), respectively, as measured with goniometry (Baseline®, HiRes® 360° 30-cm, Fabrication Enterprises Inc., White Plains, NY, USA). These configurations were applied due to mechanical constraints of the dynamometer and the objective of identical anatomical knee joint angle for knee extensors and flexors, respectively, to evaluate H/Q-ratios (Maffiuletti et al. 2007). Seat position and external lever arm lengths (distance from knee joint rotation center to point of ankle cuff attachment) were individually adjusted for each study participant to 2-cm above the lateral malleolus (Aagaard et al. 2002). Subjects were firmly strapped to the chair seat and backrest (10° reclined) at the hip and distal thigh. Individual seat and ankle cuff positions were the same at Test 1 and Test 2. All recorded muscle torques were corrected for the effect of gravity on the lower limb (Aagaard et al. 1995).

Subjects performed two submaximal warm-up contractions followed by five contractions at maximal voluntary effort for the knee extensors and knee flexors, respectively, with 1-min recovery between trials. The assessor provided strong verbal encouragement and study participants simultaneously received visual online feedback of their exerted torque (Aagaard et al. 2002; Maffiuletti et al. 2007; Fristrup et al. 2020). All participants were carefully instructed to contract as fast and forcefully as possible and maintain the contraction for 4–5 s or until otherwise instructed by the assessor (Fristrup et al. 2020). Knee joint torque was measured with a strain gauge-based force sensor and calculated into torque (model Z6FC3-200 kg, Hottinger-Baldwin Messtechnik GmbH, Darmstadt, Germany). The torque signal was sampled at 1000 Hz using the built-in dynamometer software (ARS dynamometry, S2P ltd., Ljubljana, Slovenia). Raw torque signals were then exported for subsequent processing and data analysis using custom build software script (MATLAB, MathWorks, Natick, USA). All recorded torque signals were low pass filtered using a digital fourth-order, zero-lag Butterworth filter with a cutoff frequency of 15 Hz (Aagaard et al. 2002; Bojsen-Møller et al. 2005; Fristrup et al. 2020). MVIC peak torque was defined as the highest peak torque value among the trials, while onset of torque was defined as 1% of MVIC. Trials with pre-contracting torque exceeding 1% of MVIC peak torque were discharged. For the statistical analysis of knee extensor and flexor MVIC, along with the calculation of hamstring-to-quadriceps strength ratios (H/Q-ratio), the trial with highest MVIC peak torque was chosen for analysis.

Impulse is represented by the area under the torque–time curve, calculated as ∫Torque d*t* with *t* being time (Aagaard et al. 2002). Impulse reflects the angular speed of the lower limb if it had been allowed to move freely, thus it might be the most functional measure of rapid muscle torque production in an isometric contraction (Maffiuletti et al. 2016). For the evaluation of RTD and impulse, the trial with highest impulse at 0–200 ms was selected for further statistical analysis since this measure represents the integrated time-history of contraction throughout entire time interval of 0–200 ms. Rate of torque development and impulse were calculated as the slope (Δtorque/Δtime) and the area (∫Torque d*t*), respectively, of the torque–time curve in the initial (early) time intervals (0–30 ms and 0–50 ms) and later time intervals (0–100 ms and 0–200 ms) relative to onset of contraction (Aagaard et al. 2002; Andersen and Aagaard 2006; Jordan et al. 2015; Maffiuletti et al. 2016; Palmer et al. 2021).

### Sprint performance

**Fig. 2:**
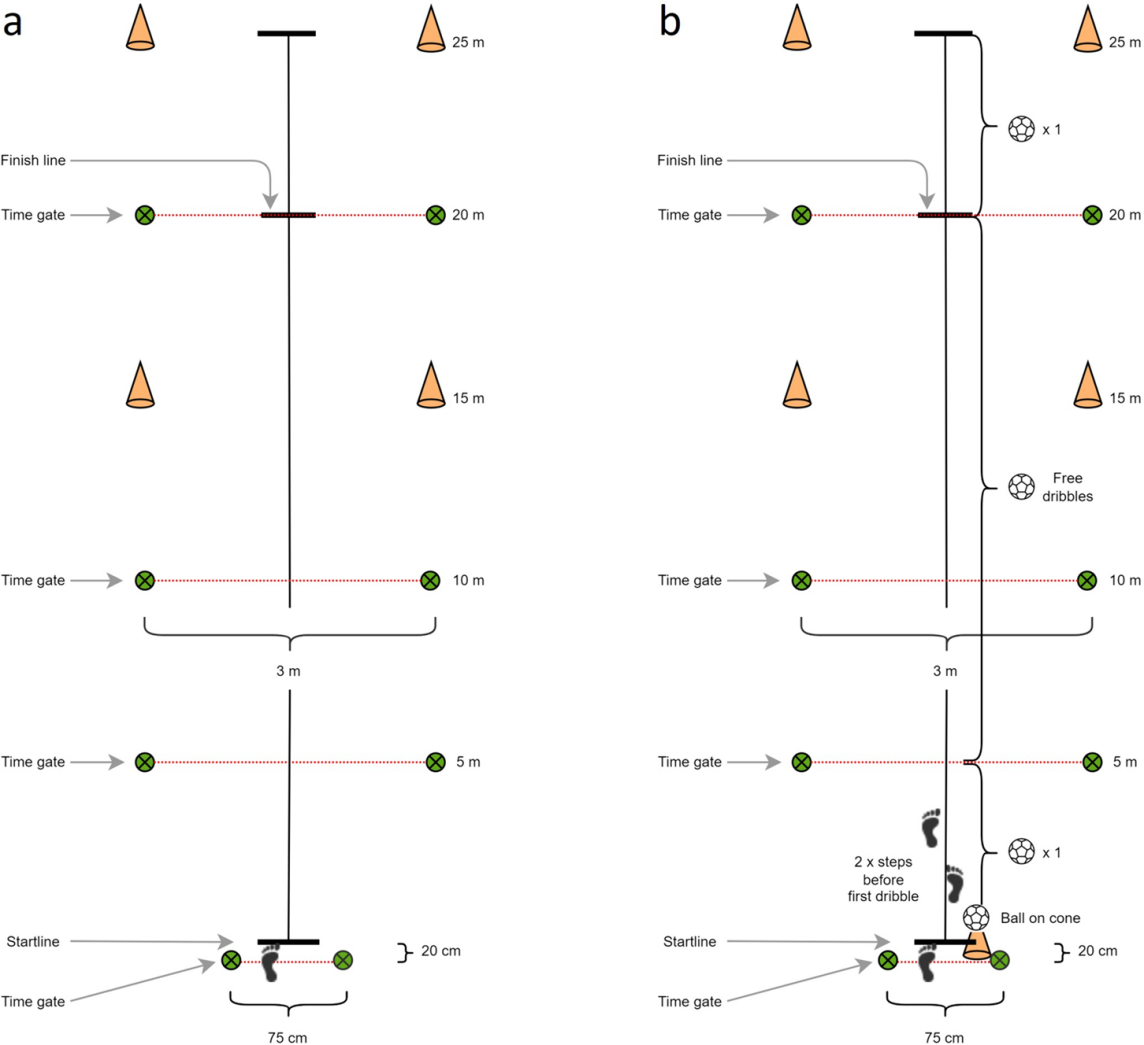
A schematic overview of the 20-m sprint test without dribbling a handball (**a**) and with dribbling a handball (**b**). Bold black lines mark start line, finish line and target finish line (25 m). Symbols of green circles with cross symbols shows photocells and yellow cones are visual markings at 15 and 25-m. In **b**, the 20-m sprint test with handball dribbles is illustrated

Sprint performance was evaluated in a 20-m maximal sprint test with target finish line at 25-m and visual markings at 5, 10, 15, 20, and 25-m (Fig. 2a). Split times were collected at 5, 10 and 20-m at hip height (75-cm), using dual-beam photocells (8 MHz Wireless Training Timer (WITTY-gates), Microgate, Bolzano, Italy). Participants started from a standing position with the front foot placed at the starting line and with the start sensor placed 20-cm behind the starting line, resulting in the beam of the start sensor being interrupted by the malleolus of the front foot. When the front foot was lifted from the start position, time would begin. Study participants performed two submaximal runs at ~ 60% and ~ 80% of maximal intensity, respectively, before completing five maximal 20-m sprints, interspaced by 1-min recovery. Subjects initiated their own start (no external cueing) after the 1-min recovery within a 15-s time window. The fastest 20-m sprint time was selected for further statistical analysis.

Subsequently, the participants engaged in five maximal 20-m sprints, while the subjects concurrently dribbled a handball, again interspaced by 1-min recovery. Standard rules of team handball were applicable, and resin was utilized. The starting position and split distances were the same as without ball. In the starting position, the ball was placed on top of a 35-cm high cone, with the participants instructed to touch the ball. Subjects performed two steps before the first dribble, releasing the ball before reaching the 5-m mark, and unrestricted dribbles were allowed between the 5- and 20-m marks (Fig. 2b). Dribbles were counted to ensure an identical number of dribbles at both test sessions. In addition, subjects were instructed to perform a single dribble between the 20- and 25-m mark, to ensure sustained ball control while crossing the 20-m finishing line.

### Statistical analysis

Data were examined for normal distribution by visual inspection of QQ plots. Paired students t-testing was applied to test differences of means between Test 1 and Test 2. Significance level was set at *p* ≤ 0.05 (two-tailed testing). Delta values (Test 2 – Test 1) exceeding ± 2.5 standard deviations were defined as outliers (Nielsen et al. 2017) and disregarded from the analysis. Test–retest reliability were calculated by Intraclass Correlation Coefficients (ICC), two-way random effect model (ICC_2,1_). ICC values ≥ 0.75 were considered excellent, 0.60–0.74 good, 0.40–0.59 moderate and < 0.39 poor (Fleiss 1986; Cohen 1988). Within-subject coefficient of variation (CV_w-s_) was calculated as standard deviation (SD) relative to the mean ($\overline{X}$) as previously described (Holsgaard Larsen et al. 2007):

$$CV_{{w - s}} = \frac{{SD}}{{\overline{X}}} \cdot 100$$

where

$$SD = \sqrt {\frac{{\sum \left( {d^{2} } \right)}}{2n}} {\text{and}}\,d = {\text{Test }} 2 - {\text{Test}} \, 1$$

$$\overline{X} = \frac{{\overline{X}_{{\text{Test 1}}} + \overline{X}_{{\text{Test 2}}} }}{2}$$

CV_w-s_ ≤ 15% were considered acceptable (Dos’Santos et al. 2019; Palmer et al. 2020). Critical difference (%) was calculated with a 90% probability in this study for each examined variable, to estimate a significant change between two consecutive measurements (i.e., pre and post) for an individual of the study population or with a similar physiological profile. Critical difference (CD) was calculated as ${\text{CD}}= Z \cdot \sqrt{2} \cdot {{\text{CV}}}_{w-s}$, in which Z denotes the percentage points of the Gaussian distribution for a two-tailed probability of 90% (*Z* = 1.65) (Hayes et al. 2014; Holsgaard Larsen et al. 2007).

## Results

### CMJ kinematics and kinetics

Countermovement jump test variables maximal vertical jump height, JH_GL_, peak and mean power, concentric work, and BCM_disp_ (E_p_, C_p_) demonstrated good-to-excellent test–retest reliability between Test 1 and Test 2 (ICC ≥ 0.70) accompanied by CV_w-s_ of 3.4 to 11.0% (Table 2). Duration of the C_p_ and LLS showed moderate-to-good reliability (ICC = 0.48–0.70) with CV_w-s_ values of 11.9 and 31.0%, respectively (Table 2). BCM_disp_ in the C_p_ was found to decrease (-8%) from Test 1 to Test 2 (*p* = 0.017). Finally, CMJ RFD 0–100 ms showed moderately strong (ICC = 0.46) reliability with a CV_w-s_ of 32.9%.

**Table 2:** Countermovement jump kinematics and kinetics

Variables	Test 1 (mean ± SD)	Test 2 (mean ± SD)	Between test *p*-value	CV_w-s_ (%)	Crit. Diff (%)	ICC	ICC *p*-value
Jump height cm, *n* = 20	28.7 ± 5.9	30.0 ± 6.6	0.101	8.1	19.0	0.85**	≤ 0.000
Jump height relative to GL cm, *n* = 19	38.3 ± 7.8	36.5 ± 7.6	0.064	8.1	18.9	0.85**	≤ 0.000
Peak power (C_p_) Watt/kg, *n* = 19	45.6 ± 6.3	45.2 ± 6.3	0.426	3.4	7.9	0.94**	≤ 0.000
Mean power (C_p_) Watt/kg, *n* = 20	24.2 ± 3.6	24.9 ± 3.6	0.201	6.9	16.0	0.88**	≤ 0.000
Work (C_p_) Joule/kg, *n* = 20	6.65 ± 1.18	6.47 ± 1.19	0.210	6.7	15.7	0.86**	≤ 0.000
RFD 0–100 ms (E_p-dec_) Nm·s^−1^/kg, *n* = 20	80.4 ± 36.6	80.6 ± 36.1	0.988	32.9	76.8	0.46*	0.022
LLS (E_p_) Nm^−1^/kg, *n* = 20	95.8 ± 43.2	102.0 ± 42.9	0.533	31.0	72.3	0.48*	0.014
BCM_disp_ (E_p_) cm, *n* = 20	29.0 ± 6.9	28.2 ± 6.8	0.407	10.5	24.4	0.81**	≤ 0.000
BCM_disp_ (C_p_) cm, *n* = 20	38.6 ± 7.2	35.7 ± 7.1*	0.017	11.0	25.6	0.70**	≤ 0.000
Duration (C_p_) ms, *n* = 20	277.9 ± 47.5	262.7 ± 47.1	0.140	11.9	27.7	0.55*	0.004

### Knee extensor and flexor muscle strength, RTD and impulse

MVIC peak torque for the knee extensors (Table 3) and flexors (Table 3) demonstrated excellent test–retest reliability (both ICC = 0.84) along with CV_w-s_ values of 6.8 and 6.0%, respectively.

**Table 3:** Knee extensor MVIC peak torque, RTD, and impulse

Variables	Test 1 (mean ± SD)	Test 2 (mean ± SD)	Between test *p*-value	CV_w-s_ (%)	Crit. Diff (%)	ICC	ICC *p*-value
MVIC peak torque Nm/kg, *n* = 19	3.16 ± 0.50	3.23 ± 0.57	0.323	6.8	16.0	0.84**	≤ 0.000
RTD 0–30 ms Nm·s^−1^/kg, *n* = 20	10.71 ± 5.87	9.55 ± 5.48	0.332	36.4	84.9	0.58*	0.003
RTD 0–50 ms Nm·s^−1^/kg, *n* = 20	13.61 ± 5.57	12.50 ± 6.10	0.407	31.6	73.7	0.50*	0.011
RTD 0–100 ms Nm·s^−1^/kg, *n* = 20	14.54 ± 3.93	13.83 ± 5.03	0.524	21.1	49.1	0.43*	0.028
RTD 0–200 ms Nm·s^−1^/kg, *n* = 20	10.93 ± 2.56	10.62 ± 2.92	0.594	16.9	39.3	0.56*	0.005
Impulse 0–30 ms Nm·s/kg (× 1000), *n* = 20	4.69 ± 2.28	4.36 ± 2.11	0.452	30.1	70.2	0.61*	0.002
Impulse 0–50 ms Nm·s/kg (× 1000), *n* = 20	15.36 ± 7.10	14.04 ± 6.96	0.385	31.5	73.6	0.56*	0.004
Impulse 0–100 ms Nm·s/kg (× 1000), *n* = 20	71.7 ± 24.1	67.5 ± 27.6	0.487	26.6	62.1	0.48*	0.015
Impulse 0–200 ms Nm·s/kg (× 1000), *n* = 20	260.0 ± 68.2	249.0 ± 82.7	0.529	21.0	49.0	0.50*	0.012

**Table 4:** Knee flexor MVIC peak torque, RTD, and impulse

Variables	Test 1 (mean ± SD)	Test 2 (mean ± SD)	Between test *p*-value	CV_w-s_ (%)	Crit. Diff (%)	ICC	ICC *p*-value
MVIC peak torque Nm/kg, *n* = 19	1.64 ± 0.25	1.67 ± 0.25	0.308	6.0	14.1	0.84**	≤ 0.000
RTD 0–30 ms Nm·s^−1^/kg, *n* = 20	3.95 ± 1.58	3.64 ± 1.68	0.391	29.6	69.0	0.52*	0.008
RTD 0–50 ms Nm·s^−1^/kg, *n* = 19	5.42 + 2.09	5.37 ± 2.34	0.901	21.2	49.4	0.73**	≤ 0.000
RTD 0–100 ms Nm·s^−1^/kg, *n* = 19	7.59 ± 1.89	7.55 ± .95	0.849	8.5	19.8	0.89**	≤ 0.000
RTD 0–200 ms Nm·s^−1^/kg, *n* = 18	5.87 ± 0.94	5.89 ± 0.89	0.865	4.8	11.2	0.91**	≤ 0.000
Impulse 0–30 ms Nm·s/kg (× 1000), *n* = 20	1.92 ± 0.63	1.80 ± 0.63	0.380	23.0	53.7	0.54*	0.006
Impulse 0–50 ms Nm·s/kg (× 1000), *n* = 19	6.00 ± 2.14	5.88 ± 2.30	0.778	20.9	48.7	0.68*	0.001
Impulse 0–100 ms Nm·s/kg (× 1000), *n* = 19	32.70 ± 10.24	32.49 ± 10.73	0.880	13.1	30.5	0.83**	≤ 0.000
Impulse 0–200 ms Nm·s/kg (× 1000), *n* = 19	136.6 ± 29.9	134.7 ± 27.5	0.591	7.4	17.4	0.88**	≤ 0.000
H/Q-ratio MVIC peak torque, *n* = 20	0.54 ± 0.08	0.52 ± 0.08	0.277	9.9	23.2	0.57*	0.004

RTD and Impulse variables obtained for the knee extensors during the early (0–30 ms, 0–50 ms) and late (0–100 ms, 0–200 ms) phase of rising muscle force demonstrated moderate-to-good reproducibility (ICC = 0.43–0.61) with CV_w-s_ values of 31.6–36.4% and 16.9–21.1% observed for early-phase and late-phase parameters, respectively (Table 3). Comparable trends were observed for contractile impulse (Table 3). Early-phase RTD for the knee flexors showed good test–retest reliability (ICC = 0.64–0.73) between Test 1 and Test 2, with CV_w-s_ values of 21.2 to 29.6% (Table 4). Late-phase RTD for the knee flexors demonstrated excellent test–retest reliability (ICC = 0.89–0.91) with CV_w-s_ values of 4.8–8.5%, respectively (Table 4). Early-phase knee flexor impulse also demonstrated good test–retest reliability (ICC = 0.54–0.68) with CV_w-s_ of 20.9–23.0%, while late-phase impulse showed excellent test–retest reliability (ICC = 0.83–0.88) with CV_w-s_ of 7.4–13.1%. Finally, hamstring-to-quadriceps peak torque ratios (H/Q) demonstrated moderate-to-good test–retest reliability (ICC = 0.64) with CV_w-s_ of 9.9%.

### Sprint performance

Sprint times at 10- and 20-m showed excellent test–retest reproducibility (ICC = 0.90 and ICC = 0.83, respectively) with CV_w-s_ values of 1.9 and 1.5%. For 5-m sprint times test–retest reproducibility was good (ICC = 0.71) with CV_w-s_ of 2.8%. Sprint testing performed while dribbling a ball showed reduced (*p* < 0.05) test–retest reproducibility compared to free sprints (ICC = 0.66–0.89) with CV_w-s_ of 3.8 to 2.0% (Table 5).

**Table 5:** Sprint performance

Variables	Test 1 (mean ± SD)	Test 2 (mean ± SD)	Between test *p*-value	CV_w-s_ (%)	Crit. diff. (%)	ICC	ICC * p*-value
*Without ball*							
5 m * n* = 19, s	0.98 ± 0.05	1.00 ± 0.05	0.086	2.76	6.45	0.71**	≤ 0.000
10 m* n* = 20, s	1.82 ± 0.09	1.82 ± 0.08	0.628	1.90	4.43	0.83**	≤ 0.000
20 m* n* = 20, s	3.27 ± 0.16	3.28 ± 0.16	0.757	1.50	3.50	0.90**	≤ 0.000
*With ball*							
5 m* n* = 19, s	1.01 ± 0.06	1.04 ± 0.07**	≤ 0.000	3.79	8.84	0.66**	≤ 0.000
10 m* n* = 19, s	1.86 ± 0.11	1.92 ± 0.13*	0.005	3.22	7.52	0.74**	≤ 0.000
20 m* n* = 20, s	3.44 ± 0.19	3.48 ± 0.20*	0.036	1.96	4.57	0.89**	≤ 0.000

## Discussion

The main findings of the present study were that maximal isometric knee extensor and flexor strength (MVIC peak torque), CMJ peak and mean power, vertical CMJ height, JH_GL_, as well as 10-m and 20-m sprint capacity showed excellent test–retest reliability (ICC = 0.84–0.94) with small intra-individual variations between test sessions (CV_w-s_ = 1.5–8.1%). Further, knee flexor late-phase RTD and impulse demonstrated good-to-excellent test–retest correlation (ICC 0.73–0.91, CV_w-s_ = 4.8–21.2). Thus, the assessment of maximal leg muscle strength, CMJ height, SSC leg muscle power, and free sprint capacity, knee flexor late-phase RTD and impulse, respectively, appears to be highly reproducible in young elite female team handball players. These observations were supplemented by signs of moderate-to-good test–retest reliability for test parameters early and late-phase RTD and impulse for knee extensor (ICC 0.43–0.61, CV_w-s_ 16.9–36.4%), early-phase knee flexor RTD, impulse and H/Q-ratio (ICC 0.52–0.73), respectively.

### CMJ kinematics and kinetics

The countermovement jump (CMJ) is a complex, 3-dimensional multi-joint movement task involving coupled stretch–shortening muscle actions (SSC) in the lower limbs, thereby potentially introducing greater variability in the kinetic output (torque, power, RFD, LLS) compared to isolated single-joint isometric muscle contractions. An accurate measuring device with high sampling frequency is advised to analyze the ground reaction force-signal (F_z_) for various kinetic metrics (Hori et al. 2009; Bishop et al. 2022). CMJ performance (jump height, JH_GL_, peak power, mean power, work BCM_disp_) in this present study demonstrated good-to-excellent correlations (ICC 0.70–0.94, CV_w-s_ 3.4–11.0%), supporting that CMJ performance on a force plate is a reliable measurement tool for investigating SSC.

Reliability studies have typically focused on jump mats, i.e., the calculation of maximal jump height from flight time (Rago et al. 2018). In the present study, jump height was determined more accurately from the vertical velocity of the BCM at the instant of toe-off, the latter determined by time integration of the F_z_ signal. Holsgaard Larsen et al. (2007) reported good-to-excellent test–retest reliability (*r* = 0.84–0.95) and CV_w-s_ values (6.8–26.9%) when elderly women (*n* = 18, aged 72.3 ± 6.6) were assessed for CMJ maximal jump height, peak power, mean power, concentric work, concentric and eccentric BCM displacement, respectively. Rittweger et al. (2004) investigated the reproducibility of peak power in CMJ testing also using force plate methodology in a mixed study population of different gender (men *n* = 14, women *n* = 22) and young to old age (24–88 years). Although observing an excellent-to-perfect test–retest reliability for peak power (*r* = 0.99), they also noted a relatively high coefficient of variation (CV = 45.4%), suggesting a high physiological variability in terms of CMJ performance when assessed across a broad age range also comprising old adults (Rittweger et al. 2004).

The present study is the first to examine the between-day test–retest reliability of lower limb SSC muscle power production and vertical jumping performance on a force plate. While multi-joint movements inherently introduce complexity, the present study demonstrate good-to-excellent test–retest reliability for a host of selected kinetic and kinematic CMJ parameters, potentially aligning with the practical relevance of these movement variables for optimizing athlete performance. Accurate and reliable evaluations of power and vertical jump performance could serve as useful tool for coaches and athletes to monitor training status and progress.

### Knee extensor and flexor muscle strength, RTD and impulse

A high reproducibility of any portable or stationary isometric dynamometer is essential to obtain precise and consistent measurements of maximal voluntary muscle strength and RTD. The results of the study support previous findings (Sarabon et al. 2013), suggesting that the present portable dynamometer provides reproducible measures of isometric knee extensor and flexor peak torque, providing researchers with a sensitive tool for detecting training-induced changes or between-limb deficits in lower limb muscle strength. The reproducibility of isometric knee extensor peak torque recordings has previously been investigated by Sarabon et al. (2013) using a portable isometric dynamometer identical to that used in the present study, demonstrating a test–retest ICC of 0.99 (Sarabon et al. 2013). In the study of Sarabon et al. (2013), knee extensor MVIC peak torque was calculated as a 1-s average obtained in the maximal stationary part of the torque–time curve. In the present study, the highest single peak torque value (1 ms time resolution) was selected for analysis, which may have resulted in higher sensitivity but also higher between-trial variability, potentially resulting in lower ICC and higher CV_w-s_.

The test–retest reliability of RTD assessed in the initial phase (early- and late-phase RTD) of rising muscle torque for the knee extensors and flexors has not previously been investigated, utilizing the present isometric dynamometer. The present observations showed a moderate-to-good reproducibility in the early-phase (0–30, 0–50 ms) RTD and impulse for the knee extensors (ICC 0.50–0.61, CV_w-s_ 30–36%) and knee flexors (ICC 0.52–0.73, CV_w-s_ 21–29%). Notably, excellent reproducibility in late-phase (0–100, 0–200 ms) RTD and impulse was observed for the knee flexors (ICC 0.83–0.91, CV_w-s_ 4.8–13.1%) with somewhat weaker reproducibility observed for the knee extensors (ICC 0.43–0.56, CV_w-s_ 16.9–26.6%). The present data suggest that the test–retest reliability of various parameters related to the expression of explosive muscle strength (RTD, impulse) may vary considerably between different muscle groups (knee extensors vs. flexors) and different time phases (early- vs. late-phase of rising muscle force). Specifically, high CV_w-s_ values (20.9–36.4%) were observed for early-phase (0–30 ms, 0–50 ms) RTD and impulse in both the knee extensors and flexors, calling for caution when interpreting these data. Notably, excellent reproducibility was observed for late-phase (0–100 ms, 0–200 ms) RTD and impulse obtained for the knee flexors, whereas somewhat weaker reproducibility was demonstrated for the knee extensors in this phase.

Research on anterior cruciate ligament (ACL) injury prevention has identified impaired knee flexor (medial hamstring) activation during side-cutting as a significant risk factor for non-contact ACL rupture (Zebis et al. 2009, 2022; Bencke et al. 2018). Further, reduced levels of knee flexor RTD relative to knee extensor RTD (RTD H/Q-ratio) may predispose for ACL injuries (Zebis et al. 2011). Consequently, it becomes imperative to ensure reliable measurements of knee flexor RTD, especially in the most initial contraction phase (0–50 ms) given that non-contact ACL rupture typically occurs in the very early time interval (17–50 ms) after foot strike (Krosshaug et al. 2007). Measurements of maximal isometric knee flexor strength is preferably performed at 60° anatomical knee angle (0° = full knee extension) to ensure maximal torque generation (De Ruiter et al. 2004; Maffiuletti et al. 2007). In part dictated by mechanical restraints of the present dynamometer, MVIC torques were obtained in the present study at ~ 50° and ~ 46° anatomical knee joint angle for the knee extensors and flexors, respectively. Higher knee extensor peak torque and RTD/impulse values could most likely have been obtained at more flexed anatomical knee angles (~ 70°), i.e., at more elongated muscle lengths (Tillin et al. 2012). The low-to-moderately strong test–retest reliability observed for the knee extensor RTD and impulse in the present study thus suggest that stronger test–retest reliability and lower CV’s may be obtained at knee joint angles closer to optimal anatomical knee joint angle, i.e., at 60–70° (Aagaard et al. 1995). Importantly, the novel findings of excellent test–retest ICC and CV values for knee flexor MVIC peak torque, late-phase RTD and impulse indicates that the mechanical properties of this muscle group can be assessed in a reproducible manner using the present portable isometric dynamometer and experimental protocol at ~ 50° anatomical knee joint angle.

### H/Q-ratios

Previous studies investigating the H/Q-ratio based on MVIC peak torque have suggested that an elevated risk of non-contact knee joint injuries may be related to low peak torque H/Q-ratios (Aagaard et al. 1998; Greco et al. 2012; Hannah et al. 2015). In the present study, the within-subject coefficient of variation in MVIC peak torque H/Q-ratio was 9.9%, which may have important implications for both clinical practice and research. The test–retest ICC for the peak torque H/Q-ratio is dependent on four different peak torque measurements and are, therefore, sensitive to even minor changes in either parameter, which may have contributed to the relatively low ICC value (0.57) observed in the present study (Aagaard et al. 1998; Greco et al. 2012; Hannah et al. 2015). The present observation of moderately strong test–retest ICC for peak torque H/Q-ratio suggests that a high degree of variability exits for this parameter, which should be considered when interpreting data based on MVIC peak torque H/Q-ratio. Nonetheless, the observation of acceptable CV_w-s_ (9.9%) indicates that the H/Q-ratio may provide a valuable instrument for researchers and practitioners to assess muscle imbalances across the knee joint, which can be used to develop individualized performance optimization and injury prevention strategies.

### Sprint performance

Excellent test–retest reliability in 10- and 20-m sprint capacity (ICC = 0.83–0.90, CV_w-s_ 1.5–1.9%) was observed when using dual-beam photocells for evaluation of split times, whereas a good reliability (ICC = 0.71, CV_w-s_ 2.8%) was noted for the present 5-m sprint split times. Thus, sprint performance can be assessed in a highly reproducible manner at short-to-medium distances (10- and 20-m), while sprint (acceleration) capacity during the initial five meters demonstrates somewhat lower yet still acceptable reproducibility. These observations support existing data on linear sprint measured with dual-beam photocells in team sport athletes (Duthie et al. 2006). As a novel observation, good-to-excellent reproducibility was observed for sprint tests including ball dribbling (ICC = 0.66–0.89, CV_w-s_ 2.0–3.8%), underlining the high technical demands of this test. These results indicate that caution should be taken when evaluating acceleration (5 m) and short (10 m) sprint with ball dribbles. Notably, however, sprint times for 5, 10 and 20-m with ball dribbling were slightly albeit significantly slower (+ 1 to 3%) at Test 2 compared to Test 1, despite performing a separate familiarization trial in the prior to actual testing. These observations suggest that more thorough familiarization procedures or a history of extended repeated testing may be necessary to ensure a high reproducibility of sprint tests involving specific technical elements such as handling a ball, cue-based changes of direction(s), and deceleration–acceleration tasks.

### Limitations

Not all variables examined in the present study could be reproduced between the test sessions. Thus, during CMJ testing, we observed a statistically significant decrease ( – 7%) in the magnitude of BCM displacement during the concentric take-off phase (BCM_disp_ [C_p_]). This observation suggest that more detailed instructions should be given on the execution of the CMJ, to ensure full plantar flexion at the instant of toe-off (i.e., during the final part of the concentric take-off phase).

## Conclusion

The portable dynamometer examined in the present study was found to provide reproducible measures of lower limb MVIC strength (peak isometric knee extensor and flexor torque), offering researchers and coaches a sensitive tool for detecting training-induced changes or between-limb deficits in thigh muscle strength.

The various measures of explosive muscle strength (RTD, impulse) revealed varying degrees of reproducibility, with late-phase (derived at 0–100 and 0–200 ms) RTD and impulse demonstrating high reliability and low CV_w-s_ values for the knee flexors. However, caution is advised in interpreting the present data on early-phase RTD of the knee extensors as well as the early and late-phase RTD data for the knee flexors due their high CV_w-s_ values.

The present data show that force plate-based CMJ testing can provide reliable insights into an athletes’ individual jumping capacity, while including reproducible measures of kinetic and kinematic variables of importance for vertical SSC jumping performance, underlining the practical relevance of these variables for optimizing athlete performance.

Sprint performance could be assessed in a highly reproducible manner at short-to-medium distances (10- and 20-m), while 5-m sprint (acceleration) capacity showed somewhat lower yet still acceptable test–retest reproducibility.

The critical difference calculated in this study may offer valuable insights for determining individual minimum change value in future intervention studies, investigating young female elite team handball players or subjects with comparable physiological profile.

This present field-based testing approach may represent a significant step toward integrating scientific rigor with the practical demands of elite athletic testing.

## Data Availability

The data that support the findings of this study are available from the corresponding author, (BF), upon reasonable request.

## Abbreviations

ACL: Anterior cruciate ligament
BCM: Body center of mass
BCM_disp_: Body center of mass displacement
BM: Body mass
CV: Coefficient of variation
C_p_: Concentric phase
CMJ: Countermovement jump
CD: Critical difference
E_p_: Eccentric phase
E_p-acc_: Eccentric acceleration phase
E_p-dec_: Eccentric deceleration phase
FAT%: Fat percentage
G: Gravitational acceleration
GRF: Ground reaction force
H/Q-ratio: Hamstring-to-quadriceps ratio
ICC: Intraclass correlation coefficients
LLS: Lower limb stiffness
MVIC: Maximal voluntary isometric contraction
RFD: Rate of force development
RTD: Rate of torque development
SMM: Skeletal muscle mass
SD: Standard deviation
SSC: Stretch–shortening cycle
V_to_: Velocity at take off
vGRF: Vertical ground reaction force
CV_w-s_: Within-subject coefficient of variation
